# Cluster randomized trial in the general practice research database: 2. Secondary prevention after first stroke (eCRT study): study protocol for a randomized controlled trial

**DOI:** 10.1186/1745-6215-13-181

**Published:** 2012-10-03

**Authors:** Alex Dregan, Tjeerd van Staa, Lisa Mcdermott, Gerard McCann, Mark Ashworth, Judith Charlton, Charles Wolfe, Anthony Rudd, Lucy Yardley, Martin Gulliford

**Affiliations:** 1 King’s College London, Department of Primary Care and Public Health Sciences, Division of Health and Social Care Research, Capital House, 42 Weston St, London SE1 3QD, UK; 2 Clinical Practice Research Datalink, Medicines and Healthcare products Regulatory Agency, London, UK; 3 School of Psychology, University of Southampton, Southampton, UK

**Keywords:** Clinical trials, Cluster analysis, Electronic health records, Feasibility studies, Stroke, Secondary prevention

## Abstract

**Background:**

The purpose of this research is to develop and evaluate methods for conducting pragmatic cluster randomized trials in a primary care electronic database. The proposal describes one application, in a less frequent chronic condition of public health importance, secondary prevention of stroke. A related protocol in antibiotic prescribing was reported previously.

**Methods/Design:**

The study aims to implement a cluster randomized trial (CRT) using the electronic patient records of the General Practice Research Database (GPRD) as a sampling frame and data source. The specific objective of the trial is to evaluate the effectiveness of a computer-delivered intervention at enhancing the delivery of stroke secondary prevention in primary care. GPRD family practices will be allocated to the intervention or usual care. The intervention promotes the use of electronic prompts to support adherence with the recommendations of the UK Intercollegiate Stroke Working Party and NICE guidelines for the secondary prevention of stroke in primary care. Primary outcome measure will be the difference in systolic blood pressure between intervention and control trial arms at 12-month follow-up. Secondary outcomes will be differences in serum cholesterol, prescribing of antihypertensive drugs, statins, and antiplatelet therapy. The intervention will continue for 12 months. Information on the utilization of the decision-support tools will also be analyzed.

**Discussion:**

The CRT will investigate the effectiveness of using a computer-delivered intervention to reduce the risk of stroke recurrence following a first stroke event. The study will provide methodological guidance on the implementation of CRTs in electronic databases in primary care.

**Trial registration:**

Current Controlled Trials ISRCTN35701810

## Background

Stroke represents a leading cause of avoidable morbidity and mortality in the UK with 130,000 stroke events per year [1]. Patients who have had a first stroke have a considerably elevated risk of a second stroke as well as increased risk of myocardial infarction, lower limb ischemia, and other vascular events [2,3]. The outcomes for those who have a recurrent stroke are generally worse than after a first stroke, with patients likely to experience increased disability and higher healthcare costs over the longer term [4]. An important objective of preventive medical care is to reduce the risk of recurrence of stroke and progression of vascular disease.

Secondary prevention after a first stroke should include lifestyle interventions to reduce overweight, increase exercise, reduce alcohol intake and smoking, as well as attention to control of risk factors for cardiovascular disease including blood pressure (BP), serum cholesterol, and antithrombotic treatment where appropriate [1,5,6]. National audits have revealed ‘major deficiencies’ in stroke secondary prevention with 40% or fewer subjects achieving a target systolic blood pressure of <140 mm Hg at 6 months post stroke [7]. Stroke secondary prevention has been specifically incentivized within the Quality and Outcomes Framework (QOF) of the general practice contract [8] with targets for treatment and control of blood pressure (<150/90 mm Hg) and cholesterol (≤5 mmol/L), antiplatelet or anticoagulant therapy for eligible patients, and flu immunization [9]. However, these targets are flexible with some 8% of patients being excluded as ‘exceptions’ and intermediate outcomes only requiring measurement within the last 15 months to less stringent targets. While the QOF strategy represents a ‘control’ intervention, a more rigorous, evidence-based intervention strategy has been recommended in the National Clinical Guidelines for Stroke from the ICSWP [1], now in their third edition [10]. These more stringent recommendations include: BP should be controlled to <130/80 mm Hg in most patients; all subjects with total cholesterol >4 mmol/L should be treated with a statin unless there is evidence of hemorrhagic stroke; and antiplatelet therapy should be prescribed where appropriate.

The responsibility for delivering effective secondary prevention, and managing long-term problems associated with stroke, lies initially with the primary care team. Considerable efforts have therefore been made to enhance the quality of preventive medical care in general practice and to increase the achievement of clinical targets for secondary prevention of stroke. The preferred design to evaluate interventions aimed at changing practitioner behavior and improve practice during contact with patients is the cluster randomized trial (CRT) [11-13]. A CRT design is appropriate when the unit of intervention is a healthcare practitioner or a healthcare organization such as a general practice. Well designed CRTs may capitalize on the effects of within-group learning, such as may occur when clinical decision support systems are deployed to primary care teams, while minimizing cross-group contamination [14]. Van Staa *et al*. [15] suggested that the utilization of large databases of electronic health records may facilitate the implementation of pragmatic trials at low cost and with reduced data collection burden for the clinician, health service, or the patient. Use of electronic patient records facilitates the identification of all patients with a specific condition registered at a particular practice [14], potentially increasing the external validity of the study findings [15]. Therefore the proposed study will use a pragmatic CRT to compare routine practice under QOF with a more rigorous secondary prevention strategy as recommended by the ICSWP.

This proposal will build on our experience of implementing a CRT to enhance secondary prevention after stroke utilizing the South London Stroke Register [16,17]. In a recent cluster trial using stroke register data to inform stroke care, Wolfe *et al*. [18] found no improvement in risk factor management at 12-month follow-up. The trial was implemented in an inner-city locality with a modest sample size and collection of outcome data collection through interaction with participants could have primed the controls about the aims of the trial potentially leading to positive lifestyle changes or social desirability bias.

The present trial aims to compare routine practice under QOF with a more rigorous secondary prevention strategy as recommended by the ICSWP. The planned trial will extend to practices throughout the UK. Individual participants will not be aware of the aims of the intervention reducing the risk of contamination.

## Methods/Design

### Objective

The primary objective of the proposed study is to evaluate whether a more intensive secondary prevention strategy, delivered at the level of general practice and based on evidence-based recommendations to control risk factors, gives improved intermediate clinical outcomes and processes of care following first stroke.

### Study design

The effectiveness of a computer-delivered intervention to promote secondary prevention of stroke will be tested in a pragmatic cluster randomized trial with two trial arms: usual care *vs.* computer-delivered recommendations for secondary prevention of stroke. The general practice is chosen as the unit of allocation because the intervention is implemented at the level of the general practice. The duration of intervention is 12 months and the first practices were allocated in April 2012.

### Study setting and population

The study will take place in family practices in England, Scotland, and Wales that contribute data to General Practice Research Database (GPRD) which is part of the Clinical Practice Research Datalink (CPRD). The age and sex distribution of the GPRD registered population is similar to the distribution of the UK population [19]. The study sample is represented by GPRD practices that provide written consent to participate in the trial, in areas that provide research governance approval of the study. Study practices are recruited through a letter of invitation sent from GPRD/MHRA. The intervention is delivered through software known as DXS, and only those GPRD practices that use DXS Point-of-Care will be eligible to participate in the trial.

### Allocation

GPRD practices will be allocated to the two trial arms in equal proportions. The allocation is by minimization controlling for region in England (North (North-East and North-West), Midlands (East and West Midlands), South-East (South-East and East of England), South-West, and London) and country in the UK (Scotland, Wales, England) and list size (number of registered patients). This list size was dichotomized for the minimization using 7,500 as the cut-point. The allocation is performed at King’s College London using anonymized practice identifiers supplied by the recruitment team at GPRD/MHRA.

### Intervention

The intervention comprises a series of electronic prompts that promote adherence with evidence-based recommendations for secondary prevention of stroke and vascular disease following the National Guidelines [5,6]. The development and implementation of the intervention was informed by qualitative research for this project [16,17] which was adapted to recent guidelines. A key element of the intervention will be a series of electronic prompts that operationalize the ICSWP recommendations. Control practices will continue with usual care. The intervention will be implemented for 12 months at each practice. A detailed description of the development and design of the electronic prompts is provided in McDermott *et al*. [20]

### Intervention implementation

Electronic prompts will be installed remotely at intervention general practices. Electronic prompts will be activated during eligible consultations. Consultations will be eligible if the patient is aged ≥18 years and is included on the practice stroke register. During consultations with patients who have previously had a stroke, primary care professionals will see the prompts which remind them of evidence-based guidelines for secondary prevention of stroke. The main focus is on targets for control of blood pressure and cholesterol levels, appropriate prescribing of aspirin and antiplatelet drugs, and enhanced recording of stroke type (ischemic or hemorrhagic). The prompts will also provide GPs with supporting information, links to evidence that supports the recommendations, and options to print patient information. The decision on whether to follow the treatment suggestions included in the prompts will be at the discretion of the GP. The GP will also be able to terminate display of the prompt at any time. At the start of the study, a letter containing instructions on the use of the prompts will be sent to general practices in the intervention trial arm for circulation to participating general practitioners. All control practices will be sent a letter reminding them to record any relevant consultation for eligible stroke patients and to notify any adverse events. Three months into the study, all intervention practices will receive an email reminder containing instructions about the prompts including aims and implementation.

### Outcome evaluation

The evaluation of outcomes will be through analysis of routinely-collected GPRD data during a defined study period, while historical information will be used to assess the baseline characteristics of the study patients. Information routinely collected into GPRD for all registered patients includes medical history, use of medicines, hospitalizations and other resource use, smoking history, laboratory tests, letters from specialists or hospitals. GPRD also now links patients in GPRD to the English Hospital Episode Statistics, with detailed information on date, duration, and reason for hospitalization. Availability of data for all registered patients has potential to minimize biases from subject selection/recruitment.

Electronic records will be included in analyses for all patients on the practice’s stroke register. Participants that were diagnosed with transient ischemic attacks only will be excluded. The primary outcome measure is the mean systolic BP after 12 months. Modeling will be used to utilize BP values measured over time during the intervention period. [21] Secondary outcomes will be mean diastolic blood pressure; mean cholesterol concentration; proportion of patients whose eligibility for anticoagulants/antiplatelet drugs is defined; proportion of eligible patients that receive anticoagulant/antiplatelet drugs; prescription adherence with prescribed medicines; occurrence and hospitalization with vascular events including TIA/stroke, myocardial infarction, new-onset angina, and mortality. In light of recent changes in guidelines for anti-platelet treatment, an additional outcome will be the proportion of patients that received clopidogrel as the first drug of choice. Recurrent stroke events will be validated through anonymized records obtained from practices. Linked HES data may be used to evaluate hospital utilization. The method of Generalized Estimating Equations will be implemented to allow for clustering by practice, providing an analytical framework for both binary and continuous outcomes [22]. Last value carried forward and multiple imputation will be used to deal with missing values.

### Process evaluation

The study will also evaluate the obstacles, barriers, and facilitators to implementation of intervention research within the GPRD. We will conduct an intervention evaluation study towards the end of the trial including both staff at GPRD and study practices via an electronic questionnaire to ensure the anonymity of practices is maintained. General practices in the ‘control’ arm of the study will only be asked to complete two questions on ‘views and opinions’. In addition, practices who agreed to a telephone interview at the time of consent for the trial will be contacted for a telephone interview at the end of the study to discuss GPs’ opinions and feedback of experiences of taking part in the trial.

### Sample size calculation

The sample size calculation is based on a comparison between the trial intervention and usual care, represented by the targets included in the quality and outcomes framework (QOF) for stroke. Thus the QOF recommends a blood pressure target for stroke patients of 150/90 mm Hg while the trial prompts, following the ICSWP guidelines, recommend a target of 130/80 mm Hg. The age-standardized incidence of first stroke is between 1.74 and 2.16 per 1,000 in England [2,23]. In 375 GPRD practices in 2006, there were approximately 14.5 first strokes per practice per year (ISAC protocol 07–027). We propose to include for analysis all subjects whose first stroke was in the 2 years preceding the intervention start date, giving about 29 subjects per practice eligible for analysis. If the standard deviation of systolic BP in these subjects is 19 mm Hg [24,25] with an ICC by general practice of 0.032 [26], with a two-sided alpha=0.05, power=0.8, then to detect a 2.75 mm Hg difference in systolic blood pressure, about 99 practices will be required with 50 receiving the active intervention. This represents an effect size of 0.14. In reality, the number of patients per practice will be variable and the coefficient of variation of practice size may be approximately 0.65 [27]. This means that the effective number of practices is approximately 83 and the detectable difference in systolic BP will be approximately 3mm Hg. Analysis of GPRD data for stroke shows a high proportion of stroke patients had blood pressure recordings in 2006 with a mean of three blood pressure records per patient.

### Ethics

The study protocol and associated documents have received approval/favorable opinion from the National Research Ethics Service Committee London - South West (REC number: 08/H0801/113) and from the R&D from participating Primary Care Trusts (PCTs) or Health Boards (HB). Protocol amendments have received REC approval and approval/favorable opinion from participating PCTs and HBs. A senior partner at each participating general practice will be asked to give written informed consent to take part in the study [27].

## Discussion

This study, together with a previously reported trial in antibiotic prescribing [28], will provide evidence concerning the feasibility of implementing CRTs using electronic databases and its effectiveness in preventing stroke recurrence and associated vascular events. The methodology developed through this project will have wide potential for application in future research with primary care electronic databases. A key output from the research will therefore be a set of methodological guidelines that will identify and analyze the component tasks of implementing a cluster trial through electronic patient records.

Cluster trials are susceptible to bias and the implementation of a cluster trial within an electronic database offers the opportunity to evaluate such biases. For example, in practices allocated to the intervention the intervention may be associated with increased recruitment rate [29]. Also, the behavior of professionals at control practices may be modified through their participation in the study even though they are not exposed to the intervention [28].

These potential biases may be evaluated by comparing number of stroke patients at intervention practices prior to and after the intervention and also comparing changes in secondary prevention activity between non-participating and participating practices.

## Trial status

Practice recruitment stage.

## Abbreviations

CRT: Cluster randomized trials; GPRD: General Practice Research Database; HB: Health boards; HES: Hospital episode statistics; ICSWP: Intercollegiate Stroke Working Party; ITT: Intention to treat; KCL: King’s College London; MHRA: Medicines and Healthcare products Regulatory Agency; NHS: National Health Service; QOF: Quality and Outcomes Framework; PCT: Primary care trusts; REC: Research and Ethics Committee.

## Competing interests

The authors declare that they have no competing interests.

## Authors’ contributions

MG conceived of the study; AD drafted the protocol; AD and MG contributed to the implementation of the study, the allocation process and the analysis plan; TvS, CW, AR, MA, LY, and JC and contributed to the design and implementation of the study and contributed to the protocol; GM was responsible for developing recruitment procedures; LM and LY were responsible for developing the trial interventions. All authors read and approved the final manuscript.
